# Creativity, brain, and art: biological and neurological considerations

**DOI:** 10.3389/fnhum.2014.00389

**Published:** 2014-06-02

**Authors:** Dahlia W. Zaidel

**Affiliations:** Department of Psychology, Behavioral Neuroscience, University of California at Los Angeles (UCLA)Los Angeles, CA, USA

**Keywords:** visual artists, brain damage, neurology, evolution, animal innovation, intelligence and creativity, artistic talent, disinhibition and frontal lobes

## Abstract

Creativity is commonly thought of as a positive advance for society that transcends the status quo knowledge. Humans display an inordinate capacity for it in a broad range of activities, with art being only one. Most work on creativity’s neural substrates measures general creativity, and that is done with laboratory tasks, whereas specific creativity in art is gleaned from acquired brain damage, largely in observing established visual artists, and some in visual *de novo* artists (became artists after the damage). The verb “to create” has been erroneously equated with creativity; creativity, in the classic sense, does not appear to be enhanced following brain damage, regardless of etiology. The turning to communication through art in lieu of language deficits reflects a biological survival strategy. Creativity in art, and in other domains, is most likely dependent on intact and healthy knowledge and semantic conceptual systems, which are represented in several pathways in the cortex. It is adversely affected when these systems are dysfunctional, for congenital reasons (savant autism) or because of acquired brain damage (stroke, dementia, Parkinson’s), whereas inherent artistic talent and skill appear less affected. Clues to the neural substrates of general creativity and specific art creativity can be gleaned from considering that art is produced spontaneously mainly by humans, that there are unique neuroanatomical and neurofunctional organizations in the human brain, and that there are biological antecedents of innovation in animals.

## Introduction

Creativity is enormously adaptive for individuals and society. Indeed, it is hard to imagine any human progress without this capacity. It is commonly defined as the introduction of something innovatively new and positive for society that goes beyond the familiar and accepted (Zaidel, 2013b). The key to the positive feature is the social aspect, namely recognition by others and adoption as the new status quo (Hodder, 1998; Simonton, 2003). Evolution appears to favor the positive social aspects of creativity (Byrne, 1998; Mithen, 1998). Bio-social pressures are thought to have shaped the evolution of the human brain, including its size and neuroanatomical and neurofunctional configurations (e.g., Dunbar and Shultz, 2007). Art is a symbolic communicative system practiced only by humans, and argued to have become a fully practiced behavior at a time when early human social groups grew in size and complexity, and communication through language and art promoted cohesion and survival.

Art is but one example where humans demonstrate the capacity for creativity. We observe it in science, engineering, technology, business, education, and countless other domains. However, most research on the brain’s underpinning of creativity applies to general creativity. It is typically measured with laboratory-constructed tasks, not specifically with art production. But most of the findings from general creativity could apply to art as well. Conversely, since art is produced spontaneously only by humans and is ubiquitously present in human societies, gaining insight into creativity through art can help understand the neural underpinning of general creativity (Creativity is a noun, as opposed to the verb “to create”, as in to produce; a produced artwork does not necessarily meet the criteria of creativity). There is enormous variability in the capacity for creativity, some individuals are hardly creative at all and others are exceptionally creative. The neural underpinning of the creativity of Newton, Einstein, Monet, Cezanne, Chagall, and Picasso, for example, remains little understood (Boden, 2013), although we have gained important insights from the study, discussion, and exploration of their behavior, life-style, and thinking (Gardner, 1994a; Miller, 2000, 2002).

The fact that humans display inordinate capacity for creativity likely reflects the unique neurological organization of the human brain (Allman, 2000; Preuss, 2011; van Essen et al., 2012; Buckner and Krienen, 2013), the cognition afforded by it (Mantini et al., 2013), the biological antecedents of innovation evidenced in animals (Laland and Reader, 2010; Kaufman et al., 2011), bio-cultural practices (Bartlett, 1928; Hagman, 2005; Kim et al., 2013), and selective evolutionary pressures (Mithen, 1998). What in the brain triggers the moment of “rising above” established knowledge, and why are some individuals exceptionally creative, are questions that are still being explored (Shamay-Tsoory et al., 2011; Barbey et al., 2013; Jung and Haier, 2013). At the same time, several creativity-related factors have already been identified, specifically brain size in innovative animals (Reader and Laland, 2002; Lefebvre et al., 2004), neurotransmitters (Manzano et al., 2010), intelligence level (Sternberg and O’hara, 2000; Reader et al., 2011; Lefebvre et al., 2013), ecological niches (Lefebvre, 2013), and personality attributes (Gardner, 1994a; Miller, 2000).

Art in all of its manifestations (visual art, music, literature, dance, theater, and more) is an important feature of human societies because it serves as a cohesive symbolic communicative system conveying cultural norms, history, ideas, emotions, esthetics, and so on. Here, a dual perspective of brain and creativity is adopted, namely the biological ancestry and the neurological underpinnings in the human brain. (1) In examining the biological aspects, animal innovations will be emphasized, while the neurological underpinnings will be gleaned from (2) consequences of brain damage as they apply to visual art productions (in artists, dementia patients, Parkinson’s patient, autistic savants), and (3) relevant comparative neuroanatomy, functional connectivity, intelligence, and neurotransmitters.

## Biological Roots of Creativity

Viewed from a biological perspective, the roots of creativity run deep and are not necessarily limited to social or communicative considerations. Rather, basic biological needs in animals such as live-or-die (dire necessity), physical energy conservation, and survival through deception might be the primary motivators for innovation. Given adaptive evolutionary processes, it is reasonable to assume that all of these have become interwoven into the underlying brain mechanisms of creativity in humans. That is, there is a deep survival motivation to communicate through art when the communicative channel of language fails following brain damage (discussed in subsequent subsections). In such neurological cases, the turning to art is itself innovative; the produced art, however, is not necessarily creative.

Changing the status quo practices through innovation is not limited to humans. The classic example is that of blue tit birds observed to steal milk from foil sealed milk bottles by punching through with their beaks (Fisher and Hinde, 1949; Hinde and Fisher, 1951). In 1921 only a few birds restricted to a small geographical radius near Southampton, England, lapped up the cream in this way but within a few decades tens of thousands of tits throughout Britain were observed. Whether or not the initial motivation for the tits was fueled by curiosity, sheer necessity (starvation), or patient observation of human behavior is difficult to disentangle. In Japan, on the island of Koshima, researchers observed a monkey spontaneously rinsing sand off of her sweet potato in the river before eating it, something that was viewed and adopted by the rest of her group (Kawamura, 1959; Kawai, 1965). The same monkey later innovated a method for washing sand off of wheat grains by first dumping them in water and then scooping them all clean from the surface. Many more innovations in animals have been described (Reader and Laland, 2003; van Schaik et al., 2006; Bouchard et al., 2007; Laland and Reader, 2010; Benson-Amram and Holekamp, 2012).

Compared to humans, however, innovations by animals are by far fewer (Laland and Reader, 2010). Nevertheless, some species have been observed anecdotally to be creative and tested experimentally (Reader and Laland, 2003; Laland and Reader, 2010); the rate of innovations is particularly high in birds and non-human primates (Lefebvre, 2013). Pigeons tested in the laboratory and in the field innovated by solving a food-reaching problem and effectively spread the new knowledge to other pigeons (Bouchard et al., 2007). In the non-human primates category, chimpanzees and orangutans are the most innovative, and among birds, it is ravens and crows (*Corvus)*; among those, New Caledonian crows are considered to be exceptionally creative (Lefebvre, 2013). Although our evolutionary pasts have diverged tens of millions years ago, avians are part of our biological inheritance. With regards to non-human primates, to whom we are closer genetically than to avians, field observations documented numerous instances in the context of deception rather than in innovative technological skills (Goodall, 1986; Byrne and Whiten, 1992). This should not be surprising given development of social interaction, interdependence, and tight hierarchy in primate groups where survival depends heavily on cunning and flexibility (Byrne and Whiten, 1992; Byrne, 2003; Byrne and Bates, 2010). Against this background, creativity in humans can be viewed as an extension of the fundamental biological survival functions of cunning and deception.

However, not all non-human primates demonstrate the ability to innovate (Byrne and Bates, 2010). A good example is that of rhesus monkeys: Eating the flesh of coconuts is a preferred food by rhesus monkeys living in the scientific refuge island of Cayo Santiago, off of Puerto Rico. However, as Marc Hauser notes (Hauser, 2003), in the 60 years that these monkeys have been observed, despite watching coconuts fall off of trees naturally, directly into man-made trash fires, where the hard shell bursts open and the inside flesh becomes available for eating, no monkey has purposefully thrown a coconut into the fires. Doing so would have introduced an innovative way to optimize access to their preferred food, the coconut flesh.

Large brain size strongly correlates with innovations in birds, particularly with brain regions known as the hyperstriatum and neostriatum, while in non-human primates the regions involve the isocortex and the striatum (Lefebvre et al., 2004), roughly equivalent to the cortical association areas in humans. These human associations areas have grown in size several folds in the human brain compared to other mammals and other primates in the course of adaptive evolution (van Essen et al., 2012; Buckner and Krienen, 2013). Meta-analytic studies in animals have found that deviations from typical behavior that enhance survival are associated with larger brains (Lefebvre et al., 2004), although the brain’s size and its relationship to larger social groups is a possibility, too, and a source of debate (Byrne and Bates, 2010). Innovation in animals is strongly related to tool use, learning, and abilities dealing with seasonal changes. Some have argued that brain size evolution in birds is linked to regions controlling behavior rather than by environmental changes (Wyles et al., 1983; Reader and Laland, 2002; Laland et al., 2010). The significance of large brain size is the amount of information it can store, the availability of axonal connectivity to access concepts, and to cognitively manipulate them in cortical regions.

Animals capable of innovations are driven by biological needs to survive, and the same needs could have been passed on to humans and are now entwined with other human-unique creativity capacities. Structural and functional brain comparisons to animals shine light on some brain areas in humans that might explain our high creativity rate. Specifically, the cortical association areas and their equivalents in innovative birds are probably important. Comparing the human brain to that of monkeys with fMRI revealed several corresponding structural and functional networks, but with two that are unique to humans (Mantini et al., 2013), that is, the left hemisphere language network and the left fronto-parietal network. Using MRI for brain structural and parcellation analyses, investigators (van Essen et al., 2012) have found larger left Sylvian Fissure, which includes the parietal operculum, and in the medial temporal cortex, the portion with the lingual gyrus and collateral sulcus (all critical in language functions); in the right side the angular gyrus and dorsomedial prefrontal region. Such asymmetries are not found in other mammals, and could be playing a functional role in human creativity.

## Neurological Underpinning: I. Observations of Brain-Damage in Visual Artists

Neurological cases of visual artists who had practiced their craft professionally prior to the brain damage can help point the way to neuroanatomical and neurofunctional underpinnings of creativity. Approximately 50 or so cases with unilateral brain damage (largely in one side of the brain, and where the etiology is commonly stroke or tumor) have by now been described in the neurological literature (Rose, 2004; Bogousslavsky and Boller, 2005; Zaidel, 2005, 2013a,c; Finger et al., 2013; Mazzucchi et al., 2013; Piechowski-Jozwiak and Bogousslavsky, 2013).

The key questions concern post-damage alterations in creativity, as well as loss of talent, or skill. A review of the majority of these neurological cases suggests that, on the whole, they go on producing art, sometimes prolifically, despite the damage’s laterality or localization (Zaidel, 2005). Importantly, post-damage output has revealed that their creativity does not increase, nor diminish (Zaidel, 2005, 2010, 2013b). Given that the damage arises unilaterally (only one or the other hemisphere), artistic creativity in the healthy brain can not simply be attributed to a single hemisphere, dedicated neural “regional center”, network, or pathway, but rather to a diffusely represented capacity in the brain. Indeed, it would further seem that creativity is highly sensitive to brain damage, more so than artistic productivity, talent, or skill.

We could speculate that in the healthy brain cognitive associative networks in the left hemisphere alone, in the right hemisphere alone, or both hemispheres working together contribute to the creative process in art. However, recent functional neuroimaging evidence based on non-artistic behavior in healthy volunteers points to greater left hemisphere involvement in creativity (Gonen-Yaacovi et al., 2013). Where do the original ideas in the artwork arise, is a complex question that researchers would like understand (Dietrich and Kanso, 2010; Heilman and Acosta, 2013; Jung and Haier, 2013). The likely answer with regards to the cerebral hemispheres is that both are functional in exceptional creativity, but with each hemisphere contributing a different facet, yet little understood, to the creativity process (Zaidel, 2013d).

Some of the artists develop techniques to compensate for loss of basic sensory, perceptual, cognitive, and motoric abilities. However, non-artists suffering from similar brain damage display the same behavioral deficits in standard clinical tests and daily life. Such artworks can be interpreted to display novelty, talent, skill, and esthetics, and they have been so interpreted (e.g., Bogousslavsky, 2005; Drago et al., 2006; Thomas-Anterion et al., 2010). However, another interpretation is that they are remnants of previously well-practiced artistic skills, not expressions of creativity *per se*. The originality of their artworks is limited in scope and breadth, and their imagination seems curtailed compared to that of healthy artists.

One example is the loss of accurate depictions of 3-dimensional objects with right parietal lobe damage (de Renzi, 1982). Hemi-neglect or hemi-inattention of the left half of space is another example. Its manifestation is expressed in incomplete painting or drawing of the left half of the canvas. In a majority of the cases, however, neglect symptoms are short lived. The presence of the neglect syndrome has been attributed to imbalance caused by the damage between intact and diseased tissue (Zaidel, 2005), as well as to an abnormal control of the healthy tissue in the left hemisphere over the right half of space (i.e., the space that is not neglected) (Kinsbourne, 1977). Since the same perceptual deficits can be found in both artists and non-artists, they do not inform us of art-specialized neural substrates.

**Figure 1:**
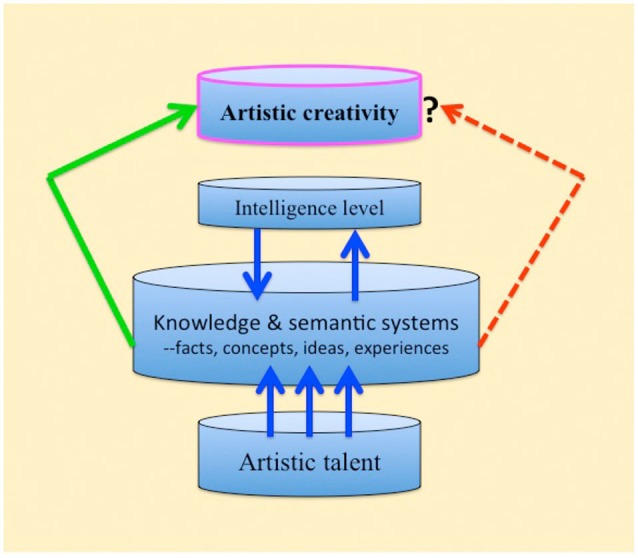
**Schematic representation of artistic creativity in health versus brain-damage (regardless of etiology)**. Artistic talent normally interacts with the knowledge and semantic systems for maximal expression of creativity. The left green arrow: the expression of creativity in the healthy brain. The right broken red arrow: with brain damage the knowledge and semantic systems are compromised and expressions of creativity are curtailed (Discussion and details in the text).

We should wonder why remarkable creativity in the art itself does not develop following brain damage, and why creativity levels remain unchanged in those artists who have practiced art prior to the damage. Compromised connectivity in the associative knowledge and semantic networks is a plausible explanation. For new ideas to originate the entire network of associations needs to be in an intact state (see Figure 1). After all, the well-known creative, influential, and important artists did not have brain damage.

Interestingly, there are published reports of neurological cases (due to stroke or head injury) of non-professional artists who commenced to practice visual art only after the brain damage occurred (Finkelstein et al., 1991; Lythgoe et al., 2005; Chatterjee, 2006; Pollak et al., 2007; Schott, 2012; Simis et al., 2013; Midorikawa and Kawamura, 2014). What can we glean from these *de novo* cases? One natural explanation is that the artistic behavior is an alternative to loss of regular language communication capacities, that is, speaking and writing. Art, too, is a communicative system, but it does not appear to be as sensitive to brain damage as language. Art conveys ideas, concepts, and emotions through different means than language (and possibly through different brain regions), and like language, it is a symbolic and referential system. Drawing and painting simply expand the communication channels between patient and caretakers, thereby enhancing survival and adaptation, much like the biological motivation to innovate in animals in order to survive.

However, published illustrations of such productions do not bespeak of creativity (e.g., Pollak et al., 2007; Midorikawa and Kawamura, 2014). Moreover, judging from the visual details depicted by the artists and the quantity of works they produce, some researchers have argued that the art has a strong obsessive-compulsive feature (Finkelstein et al., 1991; Lythgoe et al., 2005; Chatterjee, 2006; Schott, 2012; Midorikawa and Kawamura, 2014). One would have expected that the quantity alone would foster experimentation and improvement, as is the case with the prolific, culturally influential artists (Rembrandt, Goya, Van Gogh, and so on). Instead, the overall profile suggests that while brain damage does not hamper artistic expression, and allows for talent and skill to be applied effectively, it does not necessarily lead to creativity.

## Neurological Underpinning: II. Evidence from Dementia and Disinhibition

Importantly, visual artists with Alzheimer’s disease or fronto-temporal dementia (FTD), and similar brain degenerative diseases, continue to produce art well into their condition, with no obvious reduction in artistic expressions (e.g., Miller et al., 1998; Fornazzari, 2005; Crutch and Rossor, 2006). Their artistic behavior ceases when severe motoric deficits profoundly curtail their hand movements. The diffuse damage throughout the brain involves large brain areas, and this makes it difficult to attribute the artistic behavior to specific dedicated regions, pathways, or networks (see Viskontas and Miller, 2013).

A few non-artist patients with degenerative brain diseases commence to exhibit artistic behavior *de novo* following disease onset (Miller et al., 1998; Mell et al., 2003; Miller and Hou, 2004; Chakravarty, 2011; Miller and Miller, 2013; Viskontas and Miller, 2013). Interpretations have attributed the artistic behavior itself to (1) diminished inhibition of expression due to degeneration of neural pathways that normally exert inhibitory control over the cortex, i.e., frontal lobe fiber tracts running between the prefrontal cortex and the temporal lobes; and (2) neural degeneration in the left hemisphere “loosens control” over the right hemisphere, when the degenerative process originates in the left hemisphere. These interpretations assign critical roles in artistic creativity to the prefrontal cortex (e.g., Miller and Miller, 2013; Viskontas and Miller, 2013) as well as to the right hemisphere in the healthy brain (Drago et al., 2006; Heilman and Acosta, 2013). Alternative interpretations include (1) existence of life-long latent (dormant) artistic talent; (2) loss of normal language communication abilities (Zaidel, 2005). The latent artistic talent explanation is highly plausible considering that only a miniscule fraction of dementia patients exhibit spontaneous artistic behavior following disease onset. If it were as simple as loss of cortical inhibitory control, we would witness monumental artistic behavior corresponding to the number of dementia patients.

With regards to creativity, as defined here, the dementia cases exhibiting artistic behavior do not become more creative (Rankin et al., 2007; Simis et al., 2013). Consistent with this observation is a published report of 17 patients (non-artists) suffering from a frontal variant of FTD who displayed poor and diminished creativity (de Souza et al., 2010). Such findings and a recent meta-analysis of neuroimaging studies (not measuring artistic behavior) assign an important role to rostral and caudal portions of the prefrontal cortex in creativity, in general (Gonen-Yaacovi et al., 2013).

The frontal lobes have rich connections to the rest of the brain, including regions critical for memory, concept formation, and problem solving (Fuster, 2001). They exert inhibitory control on behavior, and damage in the frontal lobes often results in behavioral disinhibition, socially inappropriate behavior, and neglect of hygiene and physical appearance (Teffer and Semendeferi, 2012). Can we generalize from these behaviors, which represent deviations from accepted social norms, that patients with frontal lobe damage are creative? A recent study suggests that they become anything but creative (de Souza et al., 2010). However, insights into the neural substrates of creativity might be gleaned from decision-making research: Aron and associates (Aron et al., 2007) have suggested that the prefrontal cortex and the basal-ganglia network are involved in overcoming inhibitory neural circuitries, particularly those that impose inhibition on impulsive behavior. Fleming and associates (Fleming et al., 2010) support the notion of the prefrontal cortex and basal ganglia in status quo rejection. By inference, then, the frontal lobes are involved in some, but not all, aspects of the creative process in the healthy brain.

## Neurological Underpinning: III. Specific Neurotransmitters

Parkinson’s disease (PD), characterized by tremors and motor incoordination triggered by severe depletion of dopamine, is commonly treated with dopaminergic medication. The effects of the disease and its medication on art is revealing about the neural substrates of artistic production. Artists suffering from PD continue to produce art, despite the tremor in their dominant hand (Lakke, 1999). In both professional artists with PD and *de novo* PD artists (i.e., produce art after disease onset), a link has been found between dopaminergic medication and increased rate of artistic output. The rate is attributed to strong obsessive-compulsive components (Chatterjee et al., 2006; Kulisevsky et al., 2009; Canesi et al., 2012). This is related to other issues having to do with impulse control observed with the medication (e.g., excessive gambling, shopping, eating) and is not restricted to art production; it is a recognized side effect of dopaminergic medication (Inzelberg, 2013; Weintraub and Nirenberg, 2013).

Walker et al. (2006) reported on enhanced rate of productivity in a case of a visual artist who with dopamine medication increased his drawing and sketching activity. Similarly, Kulisevsky et al. (2009) reported on an amateur PD artist in whom increased medication led to higher rate of painting activity than previously plus a change in personal artistic technique. Schwingenschuh et al. (2010) described four successful artistic PD cases (a playwright, a fiction writer, and two professional painters) who, after dopaminergic treatment initiation, engaged in compulsive artistic output. The common denominator in all of these cases was the presence of pre-disease artistic talent; they were practicing artists. The disease condition did not obliterate their talent or creativity (nor increased it).

In addition, a link has been made between dopamine medication and newly exhibited artistic behavior (*de novo*). Schrag and Trimble (2001) described the interesting case of a PD patient who began to write high-quality poems within the first month of dopaminergic medication. Although he had not written poetry previously, his grandfather on his mother’s side was an accomplished poet. His productivity went uninterrupted and eventually he won an important poetry prize. However, only a fraction of PD patients begin to exhibit art activity after dopamine treatment, implying the treatment only releases manifestations of latent talent, not creativity *per se* (see Canesi et al., 2012; Inzelberg, 2013; Zaidel, 2013b).

The question remains, by what mechanisms dopamine acts on artistic output? An interplay of the fronto-temporal lobes and dopamine has been suggested by Flaherty (Flaherty, 2005). Similarly, Schrag and Trimble (2001) suggest that the poetry writing in their patient could have been due to the loss of inhibition over art expression (because of frontal lobe damage), as well as to the stimulation induced by dopamine and serotonin. The idea is that normally, in the healthy brain, an alteration in neurotransmitter balance together with specific functional neuroanatomical regions can contribute to artistic behavior. Obviously, artistic talent has to be in place to begin with, or else no amount of disinhibition, frontal lobe damage, or neurotransmitter imbalance would help artistically.

It should be emphasized that dopamine is a neurotransmitter involved in widely varied forms of human behaviors, including sensations of pleasure, normal motor functions, impulse control, drug addiction, concentration, gambling, and other functions (Flaherty, 2005; Schultz, 2007). In particular, de Manzano and associates (de Manzano et al., 2010) suggest that the D2 receptor in the dopaminergic system, especially in the thalamus, plays an important role in creativity in healthy individuals. The frontal lobes play a major role in executive functions such as planning ahead, working memory, attention, and cognitive flexibility (Teffer and Semendeferi, 2012). The thalamus is an important relay station in the brain sending neural signals to the frontal lobes and the rest of the brain. Although we do not yet know the specific threshold for the effects of dopamine on creativity in the healthy brain, density of D2 receptors could explain the array of normal individual variability in creativity. Whether or not levels of dopamine in conjunction with other neurotransmitters, as well as intact functions of several brain regions contribute to remarkable creativity needs to be addressed in future research.

## Neurological Underpinning: IV. Artistic Talent and Autistic Artistic Savants

Normally, artistic talent (inborn) and skill (can be taught) are enmeshed with creativity (de Moor et al., 2013; Zaidel, 2013b). Artistic talent ranges from an amateur to a professional, from a dabbler to a prolific artisan. The creative process interacts at each level of the talent continuum, providing other variables are in place, that is, we would expect increased cognitive flexibility and wide mental associations at the higher ends of the continuum. At the very minimum, remarkable artistic talent seems to be an inborn ability (de Moor et al., 2013), it is relatively rare and does not follow a normal curve in the population. Talented creative artists from the last few hundred years alone seldom bring to mind their progeny (the Brueghel family is a rare exception), and in such exceptional cases, children mostly, not grandchildren. Although the environment and culture play a role in the expression of artistic talent, the inborn aspect is a major determinant in the first place (de Moor et al., 2013).

What is the extent of interaction between talent and creativity? The special case of artistic autistic savants can provide insight. Exceptional talent for drawing and realistic spatial depictions is preserved in a tiny fraction of individuals with autism, namely in visual artistic autistic savants (Mottron et al., 2009; Treffert, 2009). The remarkable aspect of the condition is that despite extensive neurofunctional dysfunction in the brain, the neuroanatomic nature of which is little understood (Minshew and Keller, 2010; Corrigan et al., 2012), islands of drawing and painting talent are preserved. The nature of the displayed talent consists of extreme attention to details and to repeated patterns (Baron-Cohen et al., 2009). Although some cases are exceptionally prolific, and by far more talented in their art then the majority of people in the population, the evidence for creativity is weak (Sacks, 1995, 2004; Nettlebank and Young, 1996; Pring et al., 2012). However, some scholars report that they display improvisations and variations on a theme and consider this as evidence of creativity (Treffert, 2013). In any case, this constellation of high skill with questionable creativity strongly suggests that in the healthy brain creativity might be a separate process from talent.

## Brain, Intelligence, and Creativity

Obviously, there is a sliding scale for what can be considered creative. Some artists are exceptionally creative, some only moderately so, and some only a little. The definition of creativity perhaps should be modified to accommodate those that are not exceptional in this regard. Broadening the definition by loosening the conceptual boundaries of true creativity would include more individuals. On the other hand, tightening the boundaries by using the classic definition could enable researchers to arrive at a better understanding of the neural processes.

The intriguing neurological question concerns the neural events underway when creative thoughts that supersede the status quo knowledge emerge. The temporal sequences in such events, in particular, could help gain insight into the creative process (see Sawyer, 2011). Importantly, these neural substrates in any domain, art or otherwise, depend on activation of conceptual associations in semantic networks, whether they are verbal or non-verbal. These concepts represent knowledge and memory of the world acquired throughout one’s life about specific facts, skills, ideas, people, objects, actions, goals, cultural habits, and many other categories (Patterson et al., 2007). Several such networks with interconnected concepts function in the brain, within the left and right hemispheres (Jefferies, 2012; Barbey et al., 2013; Buckner and Krienen, 2013). Artistic skills improve as a result of activation of these networks. Theoretical models for how networks of connected concepts function have been proposed (Marupaka et al., 2012). Activation of remote conceptual associations, the moving away from common, stereotypical, familiar patterns is a likely scenario in creative thoughts, and healthy, intact, well-oiled connectivity (e.g., axons, myelin, synapses, neurotransmitters) is at the heart of creativity’s neural substrates.

What is missing from talented artistic autistic savants by way of creativity? Studying the behavior and life-style of highly creative people in the arts and sciences, Howard Gardner (Gardner, 1994a,b) unraveled a pattern that they had in common. A moderate intelligence level was one such factor (as well as risk taking, ability to tolerate rejection, and more). Robert Sternberg (Sternberg, 1997; Sternberg and O’hara, 2000) included intelligence level in the creativity process as well and considered it to be an important contributing factor; he also listed motivation, knowledge, personality, cognition, and the environment as important factors. The implication is that the creative process in individuals with low intelligence level is limited. If we consider the long-term knowledge and semantic system described above, and that this system has to be available for creative ideas to emerge, it is reasonable that artistic autistic savants capacity for creativity would be severely curtailed (see Figure 1).

## Conclusions

The art produced by neurological patients reveals that when brain damage is localized or diffuse, or when neurodegenerative brain disease is present, or when there is savant autism, or Parkinson’s disease, artistic depictions of the imagination are still possible. Other functions such as cognition and language could show profound impairments in the same patients. Both language and art are communicative systems that rely on symbolic and referential cognition, and yet, it is language that is more sensitive to brain damage than art. Further, some of these neurological cases exhibit artistic production capabilities not expressed previously (*de novo* artists). The remarkable aspect is the artwork production *per se*. The turning to art for communication is innovative. However, the creativity status of the artworks is a matter for debate because artistic skill (can be taught by others or can be self-taught), together with inherent talent (an inborn ability, which may have existed dormant all along) and esthetics, are all intertwined with creativity and thus too complex to fractionate and measure.

What drives these patients to produce the art in the first place? The answer might partially lie in our biological past. Animals capable of innovations are driven by survival needs, and although the creativity of humans is dependent on cognitive and semantic networks as well as on social recognition, the same biological need to survive might be part of the art production process. This would express itself in the need to communicate after brain damage impairs language abilities. While the need finds expression in producing the art, the creativity of the artwork requires intact cognitive and semantic networks. Would these patients’ imagination have been different if no brain damage were present? The answer would have to be in the affirmative. Professional practicing artists who do not suffer from these conditions display the kind of creativity that is socially influential for decades and centuries.

Access to an intact knowledge and conceptual semantic systems, healthy neural connectivity, and normal levels of neurotransmitters such as dopamine, are likely essential for creativity. The fact that only humans produce art spontaneously, that creativity is an important feature of art, and that humans are more creative than animals, all suggest that additional clues to the neural underpinnings of creativity lie in those features of the brain that are unique to humans.

## Conflict of interest statement

The author declares that the research was conducted in the absence of any commercial or financial relationships that could be construed as a potential conflict of interest.
